# Synthesis and characterization of g-C_3_N_5_/CuS/AgNPs nanocomposite as a Z-scheme photocatalyst for efficient methyl parathion degradation

**DOI:** 10.1038/s41598-026-35254-6

**Published:** 2026-01-29

**Authors:** Hakimeh Teymourinia, Hassan Abbas Alshamsi, Mehrnaz Gharagozlou, Abbas Al-nayili, Mitra Gholami

**Affiliations:** 1 Chemistry Department, Faculty of Science, Lorestan University, Khorram Abad, Lorestan Iran; 2 Department of Chemistry, College of Education, University of Al-Qadisiyah, Al Diwaniyah, Iraq; 3 Department of Nanomaterials and Nanocoatings, Institute for Color Science and Technology, PO. Box 1668814811, Tehran, Iran; 4 Research Center for Environmental Health Technology, Iran University of Medical Sciences, Tehran, Iran; 5 Department of Environmental Health Engineering, School of Public Health, Iran University of Medical Sciences, Tehran, Iran

**Keywords:** Parathion, Photocatalytic degradation, Environment, Nanocomposite, Chemistry, Environmental sciences

## Abstract

Water pollutants constitute a significant environmental concern today. Organophosphorus compounds, notably parathion, represent a critical category of water pollutants. Their extensive usage poses substantial risks to human health and the environment, necessitating their removal from water sources. This study focuses on the photocatalytic degradation of parathion, utilizing a g-C_3_N_5_/CuS/AgNPs nanocomposite synthesized through a combination of hydrothermal and ultrasonic methods. The nanocomposite was characterized using microscopic and spectroscopic techniques and demonstrated the capacity to degrade approximately 94.90% of parathion in the presence of visible light within one hour at pH 6. Parameters such as pH, initial pollutant concentration, and photocatalyst concentration were optimized. The study’s findings highlight that ^•^O_2_^−^ and ^•^OH play a predominant role in the degradation process of this contaminant.

**Supplementary Information:**

The online version contains supplementary material available at 10.1038/s41598-026-35254-6.

## Introduction

Methyl parathion, a highly potent organophosphorus compound, is extensively used in agriculture as both an insecticide and an acaricide, highlighting its crucial role in pest control strategies^1,2^. Understanding its chemical composition is fundamental to comprehending its physicochemical properties, environmental fate, and primary dispersion pathways within the ecosystem. Similar to other organophosphorus insecticides, methyl parathion functions by inhibiting acetylcholinesterase, a key enzyme in the nervous system, leading to the accumulation of acetylcholine and subsequent neurological damage^3–5^. Despite its low water solubility, which limits its accumulation in aquatic environments, methyl parathion undergoes microbial degradation, photolysis, and hydrolysis, resulting in 4-nitrophenol as a major degradation byproduct^6^. Exposure to methyl parathion may cause nausea, vomiting, weakness, and headaches. Moreover, it can induce oxidative stress and liver damage, potentially leading to severe hepatitis and renal failure^7,8^. The environmental impact of this compound is evident in its ability to cause developmental abnormalities, oxidative stress, and reduced survival rates in various organisms. Additionally, it induces significant cellular damage, such as lipid peroxidation and genotoxicity, underscoring its extensive toxicological effects^9^.

Several techniques have been developed to eliminate methyl parathion from the environment, including microbial biodegradation, Fenton^10^, chemical degradation via advanced oxidation processes (AOPs), photodegradation facilitated by sunlight and photocatalysts, adsorption using activated carbon, phytoremediation through specific plant species, and physical separation via membrane technology^11–14^. Among these, AOPs—such as photocatalysis and Fenton reactions—have gained increasing attention due to their ability to degrade persistent organic pollutants into harmless substances. These methods are effective even at low concentrations and are adaptable for treating various organic contaminants. Additionally, AOPs can be implemented under diverse environmental conditions with relatively simple configurations, ensuring broad applicability^15–17^. The aforementioned methods are less practical compared to photocatalytic approaches due to long reaction times, production of secondary pollutants, and low stability. Photocatalytic methods, on the other hand, benefit from the use of nanoparticles with high specific surface area and the synergistic effects of multi-component composites, which enhance and facilitate electron transfer, resulting in superior performance^18^. Photolysis, which utilizes light energy to break down organic pollutants into harmless byproducts, has emerged as a promising, efficient, cost-effective, and environmentally friendly technology, drawing significant interest from researchers. One of the approaches to enhancing photocatalyst efficiency is their activation using NaBH_4_, and peroxymonosulfate. This process facilitates rapid electron transfer and reduces electron–hole recombination. Moreover, by generating highly reactive sulfate radicals, the degradation efficiency of pollutants is improved. Therefore, understanding the behavior and mechanisms of chemical activators is essential for interpreting the photocatalytic process^19,20^.

The environmental viability of this method lies in its ability to degrade pollutants without generating hazardous residues^21–23^. When photons possess energy greater than the band gap of a semiconductor, electron-hole pairs (e_CB_^−^/h_VB_^+^) are generated, initiating the photocatalytic process. Highly efficient photocatalysts should exhibit effective charge separation and transfer to prevent recombination, thereby maximizing the photocatalytic reaction rate^24,25^. A review of the literature indicates that various photocatalysts have been employed to degrade parathion, including graphene oxide-Fe_3_O_4_/Bi_2_MoO_6_ nanocomposites^26^, NiO/Bi_2_MoO_6_^27^, Ag-doped TiO_2_ nanoparticles^28^, Fe_3_O_4_@SiO_2_@mTiO_2_ nano-microspheres^29^, ZnO nanostructures and their Cu-loaded composites^30^, ZnO/CuO nanophotocatalysts^31^, Cu_2_O nanoparticles^32^, MnO_2_ nanoparticles^4^, and Bi^3+^-doped TiO_2_^33^. To enhance photocatalytic efficiency, selecting an appropriate photocatalyst that optimizes reaction rates and performance is essential. Photocatalysis is employed in various applications including synthesis, hydrogen production, photoinactivation, ammonia production, etc^34^.

Graphitic carbon nitride (g-C_3_N_4_) has gained attention as a non-toxic, metal-free photocatalyst capable of utilizing visible light excitation, paving the way for environmental remediation and energy conversion applications. Its attractive properties—such as low density, excellent thermal and chemical stability, biocompatibility, and strong absorption in the visible and near-infrared regions—make it a promising photocatalyst for a variety of applications^35^. With a band gap of 2.7 eV, g-C_3_N_4_ exhibits enhanced light absorption and a favorable electronic structure for photocatalytic processes^36,37^. However, despite these advantages, g-C_3_N_4_ suffers from limited surface area and rapid charge carrier recombination, restricting its broader application. Incorporating metal or non-metal dopants into g-C_3_N_4_ can modify its electronic structure, improving charge separation and transport efficiency and thereby enhancing photocatalytic performance^38^. Nitrogen doping in g-C_3_N_4_ is a particularly effective strategy for tailoring its band structure. By modifying the primary π-π bonding framework, nitrogen doping narrows the band gap, enabling the absorption of a broader range of light wavelengths while also increasing the density of electron-trapping sites^39,40^. Although g-C_3_N_5_ offers several advantages, its narrower band gap compared to g-C_3_N_4_ limits its redox capacity and photocatalytic performance. To overcome these limitations, g-C_3_N_5_ is often combined with other semiconductors to form composite materials. These composites extend the light absorption spectrum, enhance charge separation efficiency, and improve redox potential, making them more effective photocatalysts^41^. Copper sulfide (CuS) nanoparticles are promising candidates within the chalcogenide semiconductor family due to their small band gap (ranging from 2.36 eV at 10 K to 2.22 eV at 300 K)^42,43^, strong absorption in the near-infrared region^44^, and specific surface area of 25.209 m² g⁻¹^45^. This material’s ability to absorb visible light and generate highly reactive intermediates upon illumination makes it a strong contender for photocatalytic applications. The superior mobility of charge carriers (electrons and holes) in CuS nanoparticles enhances its photocatalytic efficiency^46^. Furthermore, the unique flower-like morphology of CuS plays a crucial role in boosting photocatalytic activity by promoting charge carrier generation and separation. Notably, flower-like CuS nanoparticles exhibit superior photocatalytic performance compared to their spherical counterparts due to their enhanced light absorption, higher quantum efficiency, and increased surface area^47^. While CuS helps narrow the band gap when integrated with g-C_3_N_5_, incorporating an additional component with strong light absorption capabilities is crucial for maximizing its photocatalytic potential.

Silver nanoparticles (Ag NPs) have a band gap ranging from 3.55 to 4.02 eV, which is higher than that of bulk silver^48,49^. They possess remarkable properties suitable for photocatalysis, including efficient light absorption in both the visible and ultraviolet regions, a high surface-to-volume ratio, excellent electron mobility, and low electron-hole recombination efficiency. Additionally, their antimicrobial characteristics contribute to their diverse applications^50^. The localized surface plasmon resonance (LSPR) effect of Ag NPs enables strong light absorption and the generation of highly reactive electron-hole pairs. These photogenerated charges facilitate redox reactions, effectively degrading organic pollutants into non-toxic products^51^. This research focuses on the development of a novel Z-scheme photocatalyst composed of flower-like CuS and g-C_3_N_5_ decorated with spherical Ag nanoparticles. This innovative photocatalyst exhibits superior performance in the degradation of methyl parathion. Z-scheme photocatalysts consist of two semiconductors with different energy band gaps, forming a Z-shaped energy band structure^52,53^. In the Z-scheme photocatalytic process, an exceptional electron transfer event occurs, where electrons migrate from a semiconductor with a narrower band gap to one with a broader band gap. This unique configuration enhances light absorption, promotes efficient charge carrier separation, and improves redox potential, allowing for the effective utilization of a broad range of light wavelengths in photocatalytic applications^54^.

Therefore, in this study, a photocatalyst (g-C_3_N_5_/CuS/AgNPs) with a high specific surface area, resulting in a large number of active sites and capable of multi-electron transfer, which leads to a high degradation rate of pollutants, is used for the degradation of methyl parathion. Moreover, due to its low cost, easy preparation method, and environmental compatibility, it is an appropriate choice. The most important features of this photocatalyst include its chemical stability, resistance to self-decomposition in the reaction environment, and its high specific surface area arising from its nanoscale size^55^. In this work, a ternary nanocomposite is developed in which carbon nitride and silver nanoparticles synergistically enhance visible-light absorption, resulting in improved electron–hole separation. In contrast, anaerobic digestion is a slow, microbe-dependent process and an inefficient method for degrading pesticides. The proposed method here is rapid, stable, cost-effective, environmentally friendly, and highly efficient. On the other hand, anaerobic digestion, besides producing secondary pollutants and having low efficiency, is a lengthy process.

## Experimental

### Materials and equipment

The materials utilized in this study, including melamine (99.8%), hydrazine (30%), sulfuric acid (98%), hydrochloric acid (37%), sodium hydroxide (99.99%), and cetyltrimethylammonium (CTAB, 99%), sodium borohydride (98%), silver nitrate (99.8%), copper nitrate (99.99%), and others with analytical grade purity, were all procured from Merck and Sigma. To assess the morphology and crystalline characteristics, as well as to analyze the synthesized materials, advanced analytical techniques such as Field Emission Scanning Electron Microscopy (FE-SEM), Energy Dispersive X-Ray Spectroscopy (EDS), Brunauer-Emmett-Teller (BET) surface area analysis, Transmission Electron Microscopy (TEM), and X-Ray Diffraction (XRD) spectroscopy were employed. The pH of the solutions was meticulously adjusted using a Metrohm model pH meter. The photocatalytic degradation processes were monitored using a Shimadzu UV-Visible Spectrophotometer. Additionally, a Xenon lamp with a wavelength of less than 400 nm and an intensity of 400 W, positioned at a distance of 50 cm, was used for the effective degradation of pollutants.

### Synthesis of g-C_3_N_5_

g-C_3_N_5_ was synthesized following documented procedures. Initially, 6 g of melamine was placed in an aluminum porcelain crucible and heated in an oven to 425 °C at a rate of 20 °C/min for 12 h. After cooling, the solid was pulverized using a mortar, dissolved in 100 mL of water, and refluxed for 5 h. The resultant powder was separated by centrifugation and air-dried at room temperature. Subsequently, 1.6 g of the dried powder was dispersed in 15 mL of hydrazine, transferred to an autoclave, and maintained at 140 °C for 24 h. After cooling, the autoclave’s contents were washed multiple times with HCL and sodium hydroxide to remove any unreacted precursors. After three wash cycles, the powder was further purified with water and ethanol, then separated by centrifugation and dried at ambient temperature. The final step involved heating the synthesized powder at 450 °C for 2 h to obtain the desired g-C3N5 product.

### Synthesis of Flower-like CuS

In the synthesis of flower-like copper sulfide (CuS), 2 g of copper nitrate were dissolved in 50 mL of water, incorporating 0.1 g of g-C_3_N_5_ as a catalyst. Subsequently, 3 g of thiourea and 0.2 g of CTAB were added to the solution. The mixture was stirred for 1 h at room temperature before being transferred to an autoclave. The reaction was conducted at a temperature of 150 °C for 24 h. After completion of the reaction, the material was dried to obtain the flower-like CuS structure.

### Synthesis of g-C_3_N_5_/CuS/AgNPs nanocomposite

To synthesize the g-C_3_N_5_/CuS/AgNPs nanocomposite, 0.5 g of the g-C_3_N_5_/CuS nanocomposite was first dispersed in 50 mL of water. Subsequently, 20 mL of a 0.25 M silver nitrate solution was gradually added with continuous stirring. Following this, 15 mL of a 2 g/L sodium borohydride solution was introduced to the mixture, which caused the solution to turn black. The mixture was then stirred continuously at room temperature for 6 h. Afterward, the resultant product was separated using centrifugation and dried at 60 °C. The final product obtained was designated as the g-C_3_N_5_/CuS/AgNPs nanocomposite (Figure S1).

### Photocatalytic degradation study

The photocatalytic degradation of parathion, a significant and hazardous organic environmental pollutant, was investigated using the synthesized g-C_3_N_5_/CuS/AgNPs nanocomposite under visible light illumination. Initially, the parathion was exposed solely to visible light for one hour, followed by exposure to the synthesized nanocomposite for an additional hour to assess its degradation. Subsequently, the pollutant, in conjunction with the nanocomposite, was subjected to visible light for one hour at a pH of 6. During this period, samples were collected at 15-min intervals to monitor the photocatalytic degradation process, which was analyzed using a UV-Vis spectrometer at a wavelength of 240–550 nm, as stated in Eq. 1. All photocatalytic degradation experiments of parathion were carried out under visible light using a Xenon lamp, without any UV or simulated solar light exposure. The initial concentration of the pollutant, photocatalyst dosage, and pH were maintained as previously described.

Further, the rate constant for the photocatalytic degradation of parathion in the presence of g-C_3_N_5_/CuS/AgNPs nanocomposite under visible light was determined according to Eq. 2. The study also explored various parameters affecting the degradation process, including pH, initial pollutant concentration, and dosage of photocatalyst, types of scavengers, and the reusability of the synthesized nanocomposite, to achieve optimal degradation results.

1 $$\% {\mathrm{Degradation}} = \left( {{\mathrm{C}}_{0} - {\mathrm{C}}} \right)/{\mathrm{C}}_{0} *{\mathrm{1}}00$$

2 $${\mathrm{Ln}}\left( {{\mathrm{C}}_{0} /{\mathrm{C}}} \right) = - {\mathrm{kt}}$$

## Results and discussion

### Analysis and characterization

**Fig. 1:**
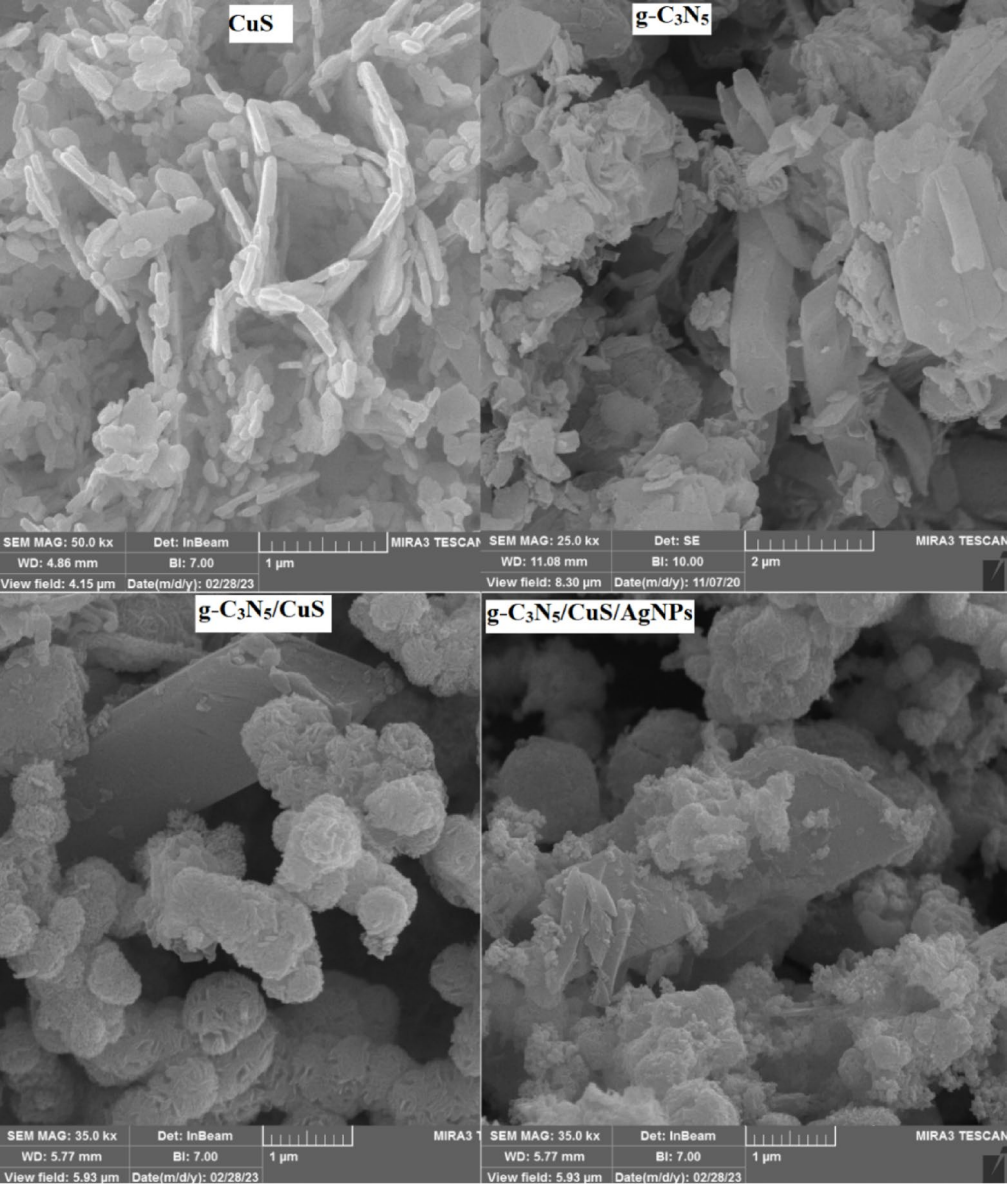
FE-SEM image of synthesis of materials.

Figure 1 presents the FE-SEM images depicting the flower-like morphology of CuS, characterized by uniform and regular spherical surfaces, which are conducive to pollutant adsorption, thereby facilitating enhanced degradation. The specific surface area of CuS, determined through BET (Figure S2) analysis, is 54.25 m²/g, highlighting its efficacy as a substrate for pollutants or other nanoparticulate entities. Additional imaging reveals the platy and rocky textures of g-C_3_N_5_, with the formation of the g-C_3_N_5_/CuS nanocomposite illustrating an amalgamation of clay nanoplate structures resembling CuS and g-C_3_N_5_. These images confirm that the integration of g-C_3_N_5_ does not compromise the structural integrity and aesthetics of the CuS nanosheets. BET analysis indicates surface areas of 49.43 m²/g for g-C_3_N_5_ and 73.12 m²/g for the g-C_3_N_5_/CuS nanocomposite. Despite the incorporation of silver nanoparticles into the g-C_3_N_5_/CuS nanocomposite, there is no significant alteration in the surface area. Nevertheless, the FE-SEM images show spherical silver nanoparticles on the surface of the g-C_3_N_5_/CuS nanocomposite, which enhance its electrical conductivity, suggesting a functional advantage in the composite’s photocatalytic activity. The specific surface area of g-C_3_N_5_/CuS/AgNPs, determined through BET analysis, is 110.22 m²/g.

**Fig. 2:**
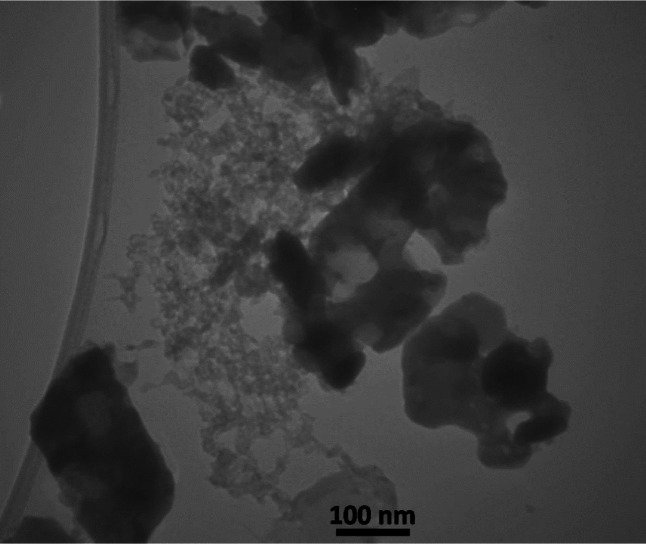
TEM image of g-C_3_N_5_/CuS/AgNPs nanocomposite.

The incorporation of these materials also leads to improved photocatalytic efficiency, resulting in an increased degradation rate of pollutants. To further evaluate the synthesized materials, band gap analysis was conducted, with the results presented in Figure S3. The band gap values for g-C_3_N_5_, CuS, g-C_3_N_5_/CuS, and g-C_3_N_5_/CuS/AgNPs are reported as 2.5, 2.0, 1.8, and 1.5 eV, respectively. These results indicate that the g-C3N5/CuS nanocomposite exhibits superior performance. The presence of AgNPs in the g-C_3_N_5_/CuS/AgNPs composite further enhances its photocatalytic activity, demonstrating the beneficial synergistic effects within the material structure. EDS (Figure S4) analysis was performed to ascertain the elemental composition of the synthesized materials, confirming the presence of carbon, copper, sulfur, oxygen, and silver, which evidences the successful synthesis of the nanocomposite and its constituents. In the g-C_3_N_5_/CuS/AgNPs nanocomposite, the elemental weight percentages were identified as follows: carbon (21.09%), nitrogen (17.41%), silver (24.97%), copper (17.30%), and sulfur (19.23%). Figure 2 displays the TEM images, where the flower-like structure of CuS is evident. Additionally, these images showcase the presence of silver nanoparticles and g-C_3_N_5_ particulates, affirming the compositional makeup. The TEM images of the g-C_3_N_5_/CuS/AgNPs nanocomposite correlate well with the FE-SEM observations, providing a comprehensive view of the nanocomposite’s morphology.

**Fig. 3:**
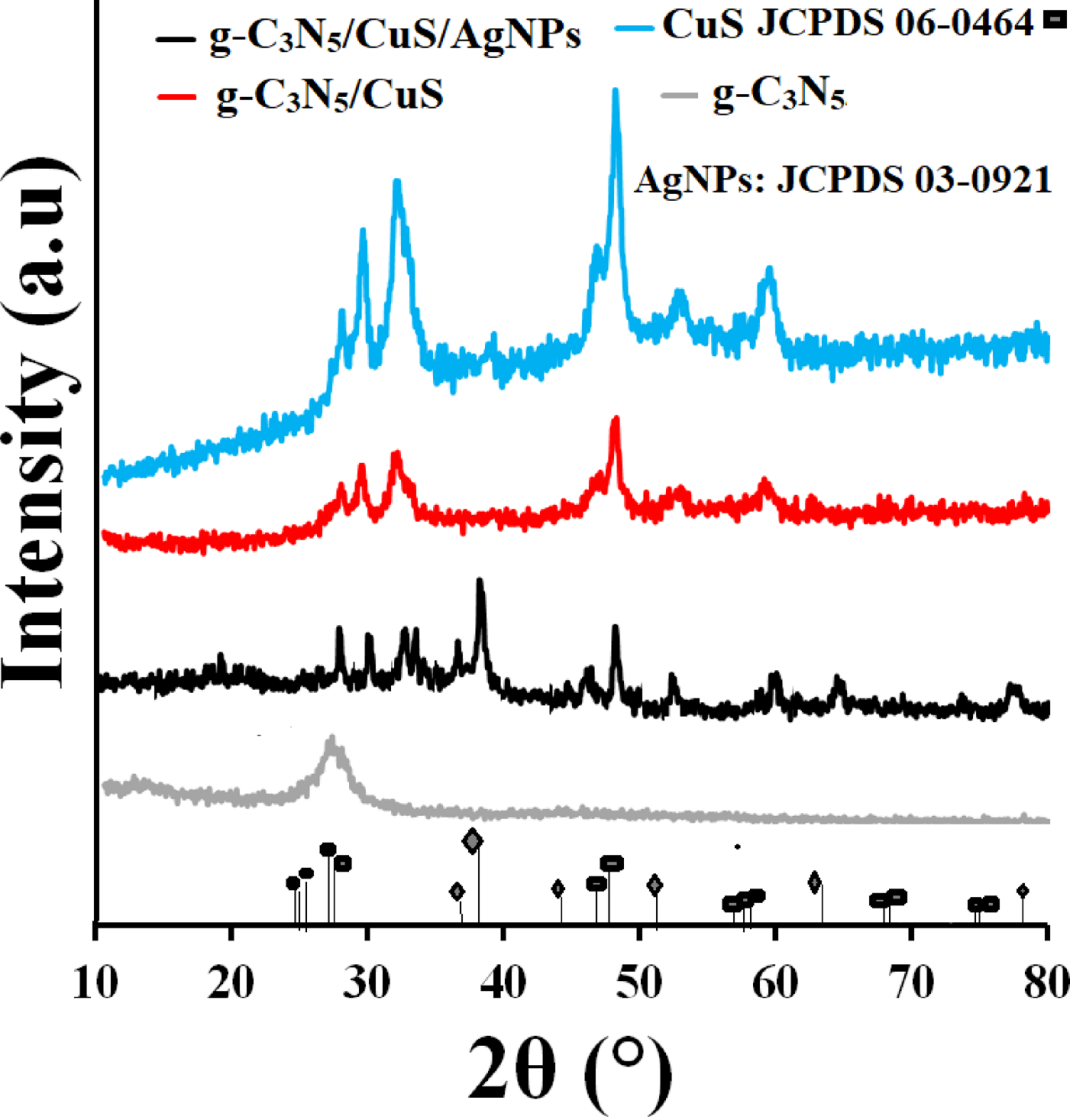
XRD pattern of material synthesis.

The XRD pattern of the synthesized materials, presented in Fig. 3, effectively demonstrates the successful synthesis of the compounds. This pattern features distinct peaks at 2θ angles of 27.89°, 29.69°, 31.9°, 33.14°, 48.19°, 53.22°, and 59.74°, corresponding to the crystallographic planes (101), (102), (103), (9006), (110), (108), and (116), respectively. Specifically, the XRD pattern of g-C3N5 shows a broad peak at 2θ = 27.59°, associated with the aromatic conjugates of this compound. In the case of the g-C_3_N_5_/CuS/AgNPs nanocomposite, additional sharp peaks are evident, supplementing those observed for CuS alone. These new peaks, found at 2θ angles of 38.19° and 64.30°, correspond to the crystal planes (111) and (220), characteristic of silver nanoparticles. This indicates the successful incorporation of silver nanoparticles within the nanocomposite, underscoring the effective synthesis of the materials in this study. The average crystal size of the g-C3N5/CuS/AgNPs nanocomposite, determined by the Scherrer method, is 29 nm. The FTIR spectrum of the g-C_3_N_5_/CuS/AgNPs nanocomposite is presented in Fig S5. The band observed in the range of 3300–3100 cm^− 1^ corresponds to the stretching vibrations of N-H and O-H groups, which are related to the g-C_3_N_5_ composition and surface hydrogen bonds. The band observed in the range of 2800–3000 cm^− 1^ corresponds to the stretching vibrations of C-H from organic groups during synthesis. The band appearing in the range of 1600–1700 cm^− 1^ is associated with the C = N double bond. The bands observed in the range of 1200–1400 cm^− 1^ correspond to the stretching vibrations of C-N and C-NH-C in the g-C_3_N_5_ network, indicating the preservation of the basic structure of nitrogen-doped polysilicate. The band found in the range of 600–800 cm^− 1^ corresponds to the vibrations of CuS and surface bonds of AgNPs. Changes in intensity and minor transitions in the ranges of 1300–1500 cm^− 1^ and 400–600 cm^− 1^ are related to hybrid interactions between g-C_3_N_5_, CuS nanoparticles, and silver nanoparticles, which may explain the formation of surface bonds or Z-scheme effects in the photocatalytic structure.

Photoluminescence (PL) analysis was used to investigate the electron–hole recombination in g-C₃N₅, CuS, and g-C₃N₅/CuS/AgNPs. As the results show, the PL spectrum (Figure S6) of g-C₃N₅ exhibits the highest intensity, indicating a high recombination rate of electron–hole pairs, which consequently limits its photocatalytic performance. In contrast, the lowest PL intensity belongs to the g-C₃N₅/CuS/AgNPs nanocomposite, suggesting a significantly reduced recombination rate and therefore superior photocatalytic activity. This enhanced charge separation in the nanocomposite arises from the heterojunction formed between nitrogen-rich graphitic carbon nitride and flower-like CuS, while the incorporated Ag nanoparticles act as electron sinks. These combined effects facilitate more efficient charge transfer and effectively suppress rapid recombination^56^.

The Nyquist plots of g-C₃N₅, CuS, and g-C₃N₅/CuS/AgNPs recorded in the electrochemical probe solution are presented in Figure S7. As the results show, the g-C₃N₅/CuS/AgNPs exhibits the lowest charge-transfer resistance, indicating its highest electrical conductivity and fastest charge-transfer rate. Therefore, sample g-C₃N₅/CuS/AgNPs demonstrates superior photocatalytic performance^57^.

## Study of the performance of g-C_3_N_5_/CuS/AgNPs nanocomposite in the photocatalytic degradation of parathion

**Fig. 4:**
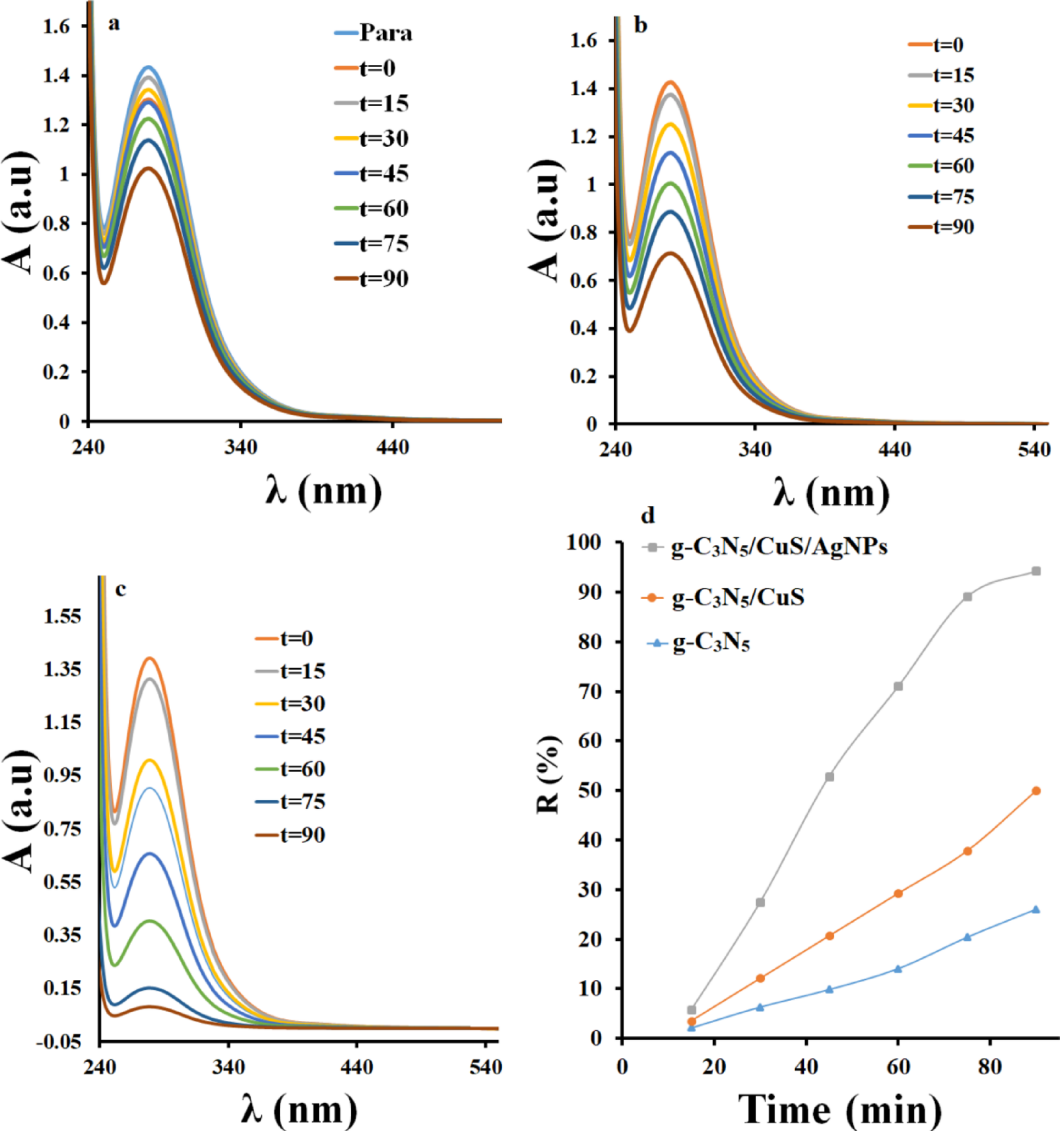
Partition absorption spectra in presence (**a**) g-C_3_N_5_, (**b**) g-C_3_N_5_/CuS and (**c**) g-C_3_N_5_/CuS/AgNPs and (**d**) the percentage of degradation in the presence of synthesized materials.

Figure 4 illustrates the degradation percentage of parathion under various conditions. The results indicate that in the presence of light alone, only 2% of parathion degrades within one hour. Conversely, in the presence of the synthesized nanocomposite, approximately 0.2% of the pollutant is degraded, suggesting that the nanocomposite alone does not significantly affect parathion degradation. Subsequently, the photocatalytic degradation of parathion was examined in combination with light and each synthesized material. The findings reveal that the nanocomposites g-C_3_N_5_, CuS, and g-C_3_N_5_/CuS/AgNPs degrade parathion by 25%, 50%, and 94.90%, respectively, within one hour, highlighting the superior degradation efficacy of g-C_3_N_5_/CuS/AgNPs. The enhanced degradation performance with g-C_3_N_5_/CuS/AgNPs is attributed to the synergistic effect of its components. None of the materials alone could achieve satisfactory parathion degradation. g-C_3_N_5_, enriched in nitrogen with an optimal N: C ratio, exhibits a smaller band gap and better conductivity, thus enhancing photocatalytic activity. Additionally, nanoplate structures like CuS increase the surface area available for contaminant adsorption^58^. The incorporation of silver nanoparticles in the nanocomposite enhances conductivity, reduces the band gap, and hinders rapid electron/hole recombination, leading to improved photocatalytic activity in degrading parathion. The g-C_3_N_5_/CuS/AgNPs nanocomposite’s larger surface area compared to the other materials further facilitates the photocatalytic degradation of parathion. This structural advantage, coupled with a smaller band gap, contributes to the nanocomposite’s effectiveness. Figure S8 presents the rate constants for the photocatalytic degradation of parathion with each synthesized material. These results demonstrate that g-C_3_N_5_/CuS/AgNPs has a higher degradation rate constant than the other materials, confirming its superior efficiency in degrading parathion.

The graph of the logarithmic changes in the initial concentration of parathion versus its concentration over different time intervals illustrates the degradation kinetics of this pesticide (Figure S8). As time progresses, the concentration of parathion decreases, indicating its breakdown in the environment or through a specific treatment process. The logarithmic scale allows for a clearer visualization of the rate of degradation, showing a linear trend if the degradation follows first-order kinetics. This suggests that the rate of concentration decrease is proportional to the concentration of parathion remaining at any given time. Analyzing the slope of the line can provide insights into the rate constant of the degradation process, which is crucial for understanding the persistence and environmental impact of parathion. Overall, this graph serves as an important tool for assessing the efficacy of methods used to mitigate parathion contamination.

### The effect of pH

**Fig. 5:**
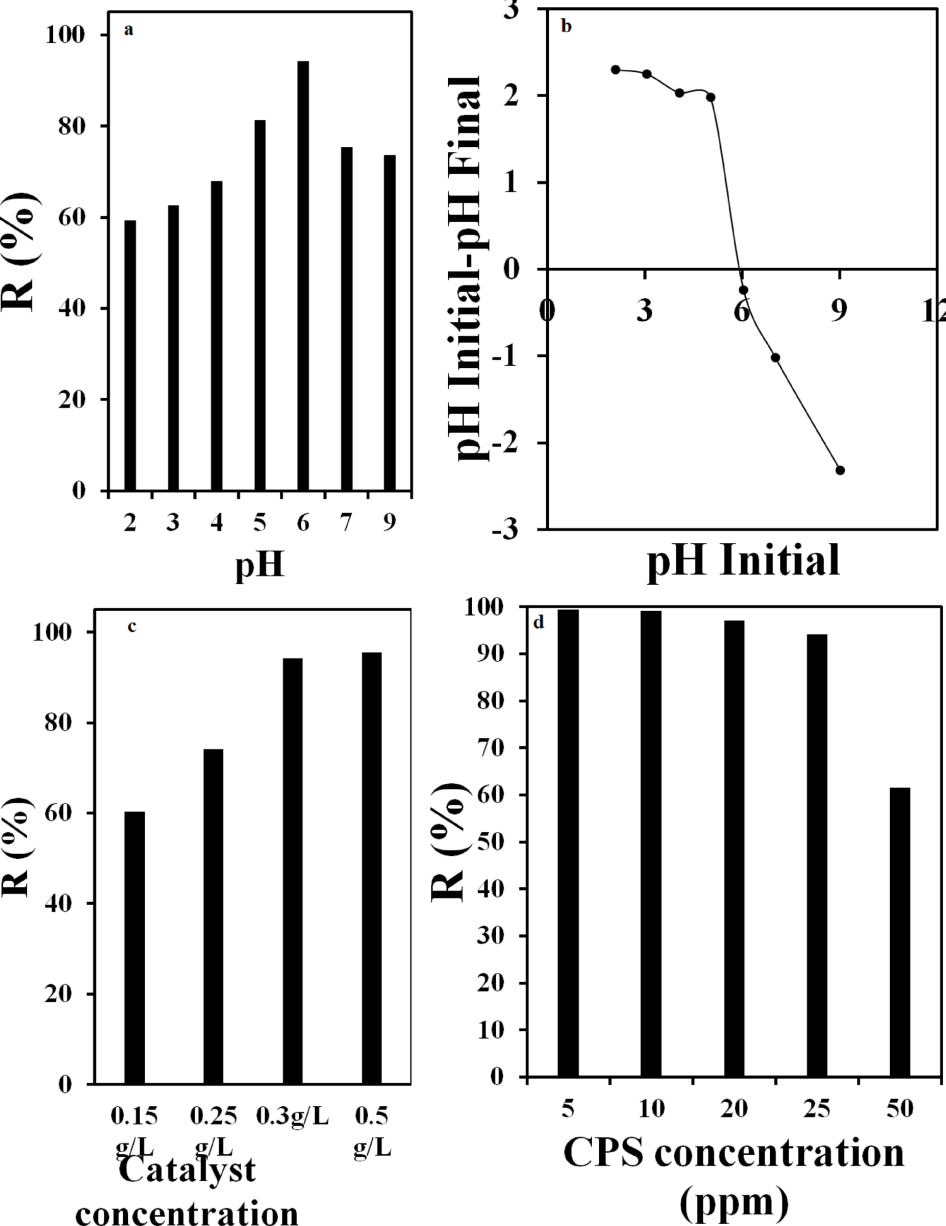
Effect of (**a**) pH, (**b**) pH_ZPC_, (**c**) nanocomposite concentration and (**d**) Initial concentration on the degradation process.

Pesticide wastewater can vary in pH when discharged, prompting an investigation into how initial pH levels affect the degradation rates of parathion, which were explored from pH 2 to 9. Figure 5a demonstrates that the degradation rate using g-C3N5/CuS/AgNPs nanopowders peaked at pH 6, following an increase from pH 3 and a subsequent decrease at higher pH values. This peak represents the optimal pH condition for the experiments and was selected for further studies. A comparative study on parathion photocatalytic degradation in g-C3N5/CuS/AgNPs noted that initial pH variations had little impact on total organic carbon conversion from pH 2 to 9. This discrepancy might be attributed to the different physicochemical characteristics of the photocatalysts and the substrate concentration on their surfaces, affecting the degradation pathways. The zero-point charge (ZPC, Fig. 5b) for semiconductor particles is the pH where equal numbers of protonated and deprotonated groups exist; for g-C3N5/CuS/AgNPs nanoparticles, this is between pH 5.4 and 5.8, leading to a negatively or positively charged surface at higher or lower pH levels, respectively. Detailed adsorption behavior of parathion on the g-C3N5/CuS/AgNPs surface was anticipated by analyzing the point charges. The balance between positively and negatively charged atoms, with significant positive charges on the P and N atoms, influences this behavior. Consequently, at a pH level of 6, slightly above the pH ZPC, the photocatalyst surface becomes negatively charged, favoring parathion adsorption and enhancing photocatalytic degradation efficiency.

### Influence of nanocomposite concentration on parathion degradation

Figure 5c presents the impact of various concentrations of g-C3N5/CuS/AgNPs nanocomposite on the photocatalytic degradation of parathion. The data indicate that the parathion degradation percentage increases with the nanocomposite concentration. Specifically, as the nanocomposite concentration rises from 0.15 to 0.5, the available adsorption sites for parathion also increase. However, at concentrations above 0.3, the aggregation of the nanocomposite leads to fewer accessible sites for the pollutant, thereby diminishing the degradation efficiency. Consequently, a nanocomposite concentration of 0.3 was identified as the optimal level for efficient photocatalytic degradation of parathion.

### Exploring the impact of parathion’s initial concentration on the degradation process

The study examined the photocatalytic degradation of parathion at various initial concentrations. Experiments were conducted with parathion concentrations of 5, 10, 20, 25, and 50 ppm in the presence of 0.3 g/L of g-C_3_N_5_/CuS/AgNPs nanocomposite at pH 6, under visible light exposure. The degradation percentages for parathion at these concentrations were found to be 99.5%, 99.1%, 97.2%, 94.2%, and 61.6%, respectively. The findings indicate that the degradation efficiency remains high up to a concentration of 20 ppm of parathion; beyond this, the degradation rate decreases. This reduction is attributed to the limited availability of active sites on 0.3 g/L of the nanocomposite for binding, leading to competition for these sites as the concentration exceeds 20 ppm, thereby decreasing the degradation efficiency. Thus, an optimal concentration of 25 ppm was determined for the photocatalytic degradation of parathion using g-C_3_N_5_/CuS/AgNPs nanocomposite (Fig. 5d).

### Exploring the influence of different scavengers and reproducibility

**Fig. 6:**
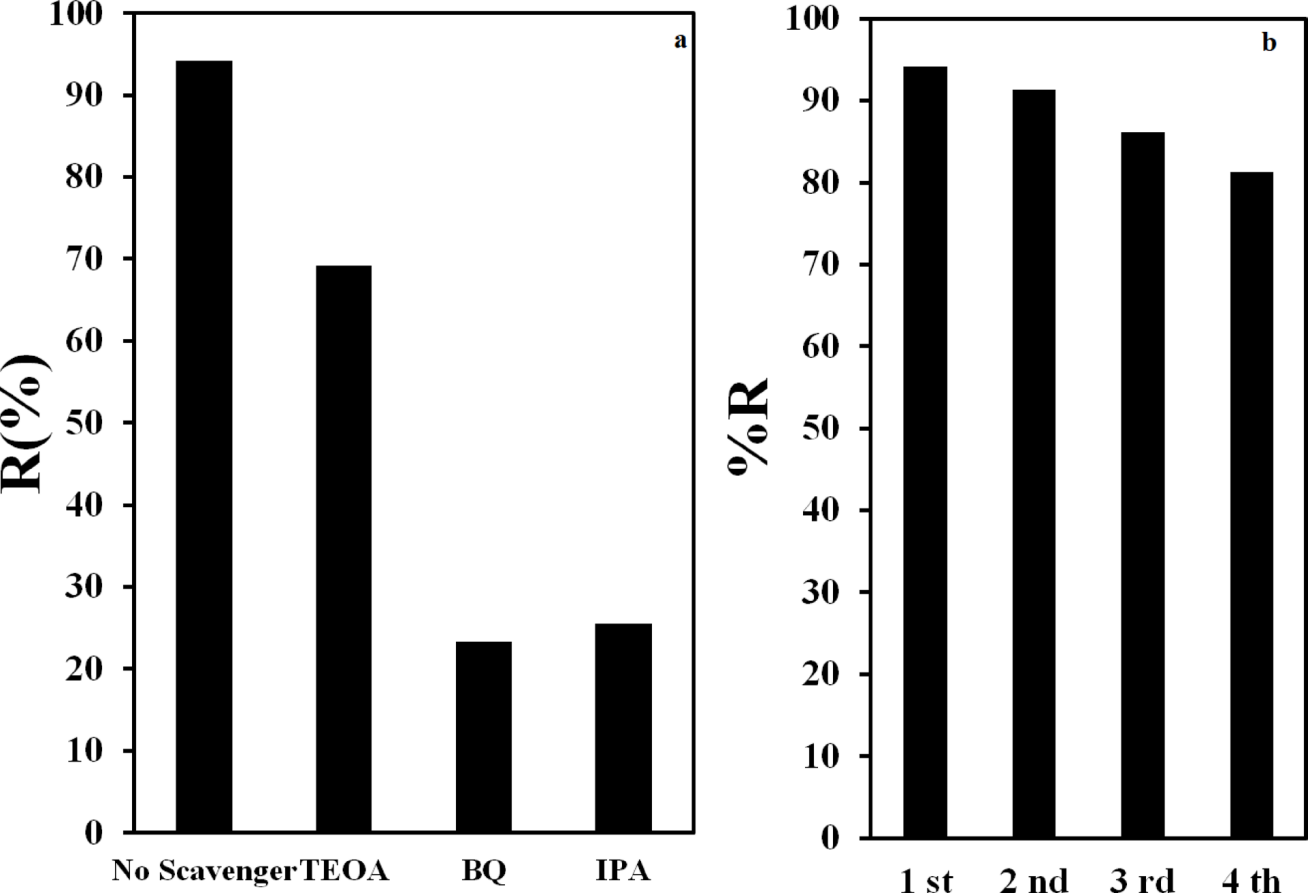
(**a**) Examination of various scavengers in the degradation of parathion and (**b**) investigation of the reuse of the synthesized nanocomposite in the degradation of parathion.

To elucidate the crucial factors in the photocatalytic degradation of parathion, various scavengers were employed. Specifically, triethanolamine (TEOA), isopropyl alcohol (IPA), and benzoquinone (BQ) were used to investigate the roles of h^+^, °OH, and °O_2_^−^, respectively. Figure 6a compares the photocatalytic degradation of parathion with and without these scavengers. The findings reveal that the presence of TEOA resulted in minimal alteration to the rate constant and degradation percentage of parathion, while IPA and BQ led to the most significant changes, indicating that ^•^OH and h^+^ play pivotal roles in photocatalytic degradation (Fig. 5a). Additionally, the reproducibility of the synthesized nanocomposite was assessed through multiple cycles of parathion degradation. The results indicated that after four cycles of use, the g-C_3_N_5_/CuS/AgNPs nanocomposite retained 81.3% of its initial efficiency in degrading parathion, demonstrating its robust performance and high reproducibility in the photocatalytic degradation process (Fig. 6b). According to the literature and published studies, ternary photocatalysts maintain their structural integrity during repeated use; therefore, it can be concluded that the synthesized nanocomposite possesses good stability^59^. A comparison to evaluate the performance of previously reported photocatalysts and the present work for the degradation of methyl parathion is provided in Table S1. As the results indicate, the degradation efficiency of this photocatalyst occurs in a shorter time.

### Study of the mechanism

**Fig. 7:**
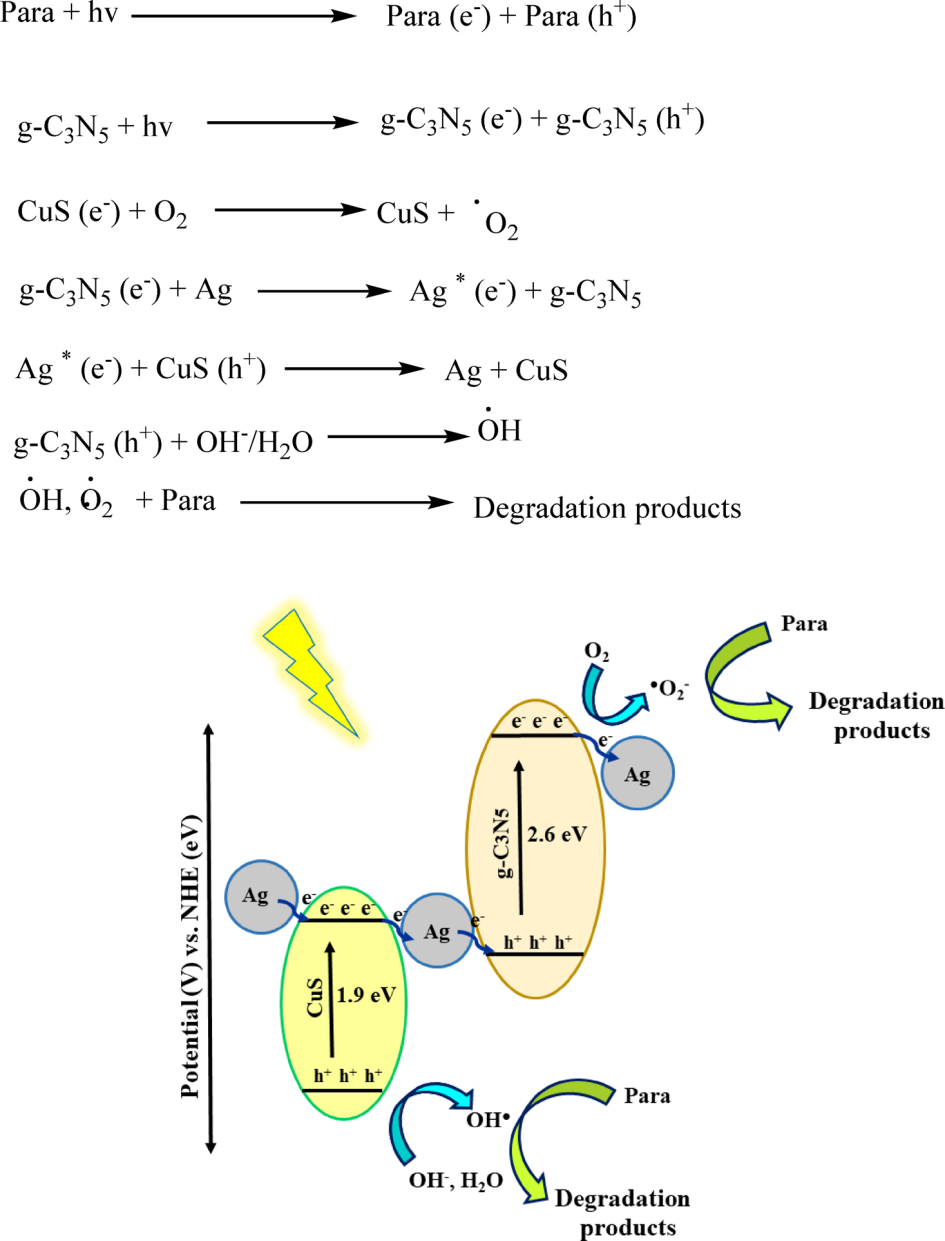
The schematic of the parathion degradation process.

The g-C_3_N_5_/CuS/AgNPs nanocomposite exhibited enhanced photocatalytic activity in the degradation of parathion, as evidenced by photocatalytic testing. To investigate the charge transfer mechanisms contributing to this improved performance, a band energy diagram of the g-_C3N5_/CuS/AgNPs nanocomposite was constructed. Analysis of Tauc plots and the VB and CB energy levels of g-C_3_N_5_ and CuS, determined through Mulliken electronegativity calculations, helped identify their band positions. It was proposed that a standard type-II heterojunction or a Z-scheme could form between the components, as shown in Fig. 7. Upon excitation in a type-II heterojunction configuration, electrons from the CB of CuS could transfer to the CB of g-C_3_N_5_ via AgNPs, with no hole transfer from the CuS VB to the g-C_3_N_5_ VB. Moreover, the generation of ^•^OH radicals in the CuS VB is improbable due to its more negative potential compared to the oxidation potentials required for ^•^OH formation. Nevertheless, studies with various scavengers showed that ^•^OH radicals significantly influence photocatalytic activity, especially in the presence of AgNPs, suggesting that AgNPs modify the charge transfer pathway on the surface of g-C_3_N_5_ and at CuS interfaces. This modification leads to the possibility of forming an all-solid-state Z-scheme heterojunction among CuS dendrites, AgNPs, and g-C_3_N_5_, where AgNPs facilitate rapid electron transfer between CuS and g-C_3_N_5_. Under visible light, the built-in electric field hinders electron flow directly from CuS to g-C_3_N_5_, favoring a Z-scheme mechanism. In this scheme, electrons excited to the CB of g-C_3_N_5_ are transferred to AgNPs and then to the VB of CuS, enabling the recombination of electrons from g-C_3_N_5_ with holes from CuS, while the strong redox potential of the g-C_3_N_5_ VB is maintained. Consequently, electrons in the CB of CuS react with adsorbed O_2_ to form ^•^O_2_^−^ radicals, and holes in the g-C_3_N_5_ VB interact with adsorbed OH or H_2_O to generate ^•^OH radicals. These reactive species facilitate the decomposition of surface-adsorbed parathion. Additionally, Ag^+^ ions may be reduced and deposited on the CuS surface during photodeposition, and the surface plasmon resonance (SPR) of AgNPs could further enhance photocatalytic activity by promoting visible light absorption and facilitating hot electron injection into the CB of g-C_3_N_5_. The presence of intrinsic or defect-induced vacancies in the g-C₃N₅/CuS/AgNPs nanocomposite is expected to further enhance charge separation and reactive species generation, contributing to the observed high photocatalytic performance^34,60^.

## Conclusion

In this study, the photocatalytic degradation of parathion, a significantly hazardous pollutant across various sectors, was achieved using the g-C_3_N_5_/CuS/AgNPs nanocomposite. The high surface area (110.22 m²/g) and low band gap (1.5 eV) of the nanocomposite contributed significantly to its efficiency. Characterized by its high surface area and low band gap, the g-C_3_N_5_/CuS/AgNPs nanocomposite exhibited exceptional efficiency in degrading parathion, achieving a 94.2% degradation rate at pH 6 with 0.3 g/L of the nanocomposite under visible light, at a parathion concentration of 25 ppm. ^•^O_2_^−^ and h^+^ were identified as key contributors to the degradation process of parathion. The robust performance of the g-C_3_N_5_/CuS/AgNPs nanocomposite in successive degradation cycles underscores its potential as a sustainable solution for pollutant degradation. The nanocomposite maintained high photocatalytic performance over four successive cycles, indicating excellent recyclability and stability. Furthermore, the nanocomposite’s straightforward and cost-effective synthesis process adds to its appeal, positioning the methodology developed in this study as a promising and accessible approach for the removal of water pollutants like parathion from the environment. This study highlights the need for deeper mechanistic investigation of charge-transfer pathways, suggesting that future work should focus on optimizing interfacial engineering to further enhance photocatalytic performance.

## Supplementary Information

Below is the link to the electronic supplementary material.

Supplementary Material 1

## Data Availability

Data is provided within the manuscript or supplementary information files.
