# Supplementary material for: Synthesis and characterization of g-C3N5/CuS/AgNPs nanocomposite as a Z-scheme photocatalyst for efficient methyl parathion degradation
Source: Sci Rep. 2026 Jan 29;16:6619. doi: 10.1038/s41598-026-35254-6 (PMC12913782; doi:10.1038/s41598-026-35254-6)
Supplement: Supplementary file 1 — Supplementary Material 1 [file 41598_2026_35254_MOESM1_ESM.docx]

**Preparation and characterization of g-C_3_N_5_/CuS/AgNPs nanocomposite as a photocatalyst of the Z-scheme for methyl parathion degradation**

Hakimeh Teymourinia^1^, Hassan Abbas Alshamsi^2^, Mehrnaz Gharagozlou ^3^, Abbas Al-nayili^2^, Mitra Gholami^4,5*^

*^1^Chemistry Department, Faculty of Science, Lorestan University, Khorram Abad, Lorestan, Iran*

*^2^Department of Chemistry, College of Education, University of Al-Qadisiyah, Al Diwaniyah, Iraq*

*^3^Department of Nanomaterials and Nanocoatings, Institute for Color Science and Technology, PO. Box 1668814811, Tehran, Iran*

*^4^Research Center for Environmental Health Technology, Iran University of Medical Sciences, Tehran, Iran*

*^5^Department of Environmental Health Engineering, School of Public Health, Iran University of Medical Sciences, Tehran, Iran*

****Corresponding author: E-mail address:*** [*gholamim@iums.ac.ir*](mailto:gholamim@iums.ac.ir)

Tabel S1. A comparative study of the performance of different photocatalysts in the degradation of methyl parathion

| photocatalysts | Time (min) | Degradation percentage (%) | Ref |
| --- | --- | --- | --- |
| oxide-Fe_3_O_4_/Bi_2_MoO_6_ | 120 | 90 | [[1](#_ENREF_1)] |
| NiO/Bi_2_MoO_6_ | 120 | 95 | [[2](#_ENREF_2)] |
| Ag/TiO_2_ | 120 | 99 | [[3](#_ENREF_3)] |
| Fe_3_O_4_@SiO_2_@mTiO_2_ | 100 | 85 | [[4](#_ENREF_4)] |
| ZnO/Cu | 80 | 99 | [[5](#_ENREF_5)] |
| ZnO/CuO | 60 | 100 | [[6](#_ENREF_6)] |
| Cu_2_O | 600 | 87 | [[7](#_ENREF_7)] |
| MnO_2_ | 360 | 80 | [[8](#_ENREF_8)] |
| Bi^3+^-doped TiO_2_ | 120 | 95 | [[9](#_ENREF_9)] |
| g-C_3_N_5_/CuS/AgNPs | 90 | 94.20 | Our work |

*
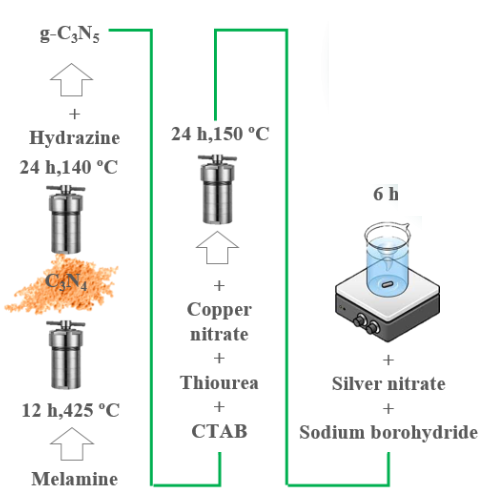
*

Fig S1. Schematic Synthesis of g-C3N5/CuS/AgNPs


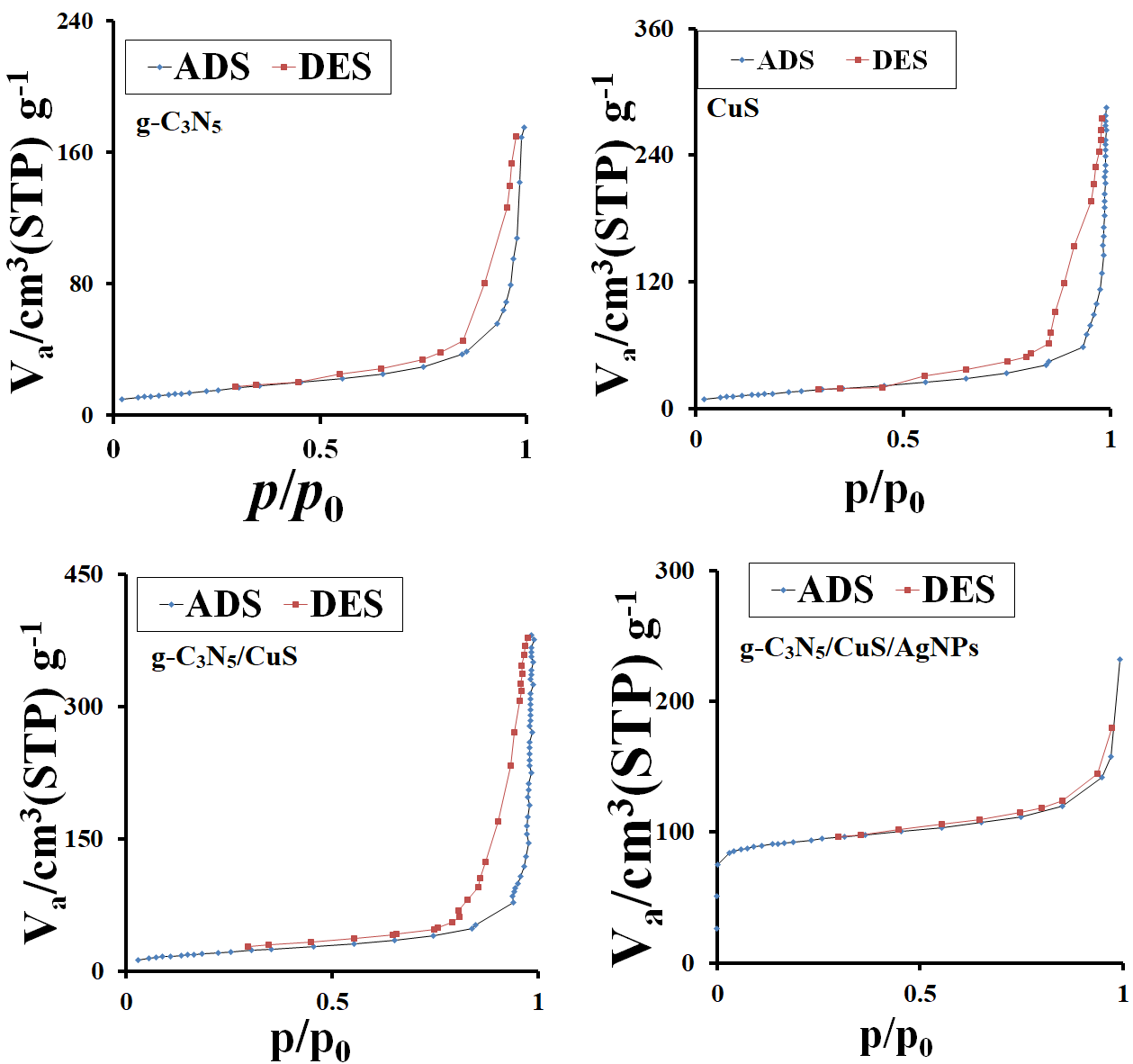


Figure S2. Adsorption/desorption isotherm of synthesis of materials


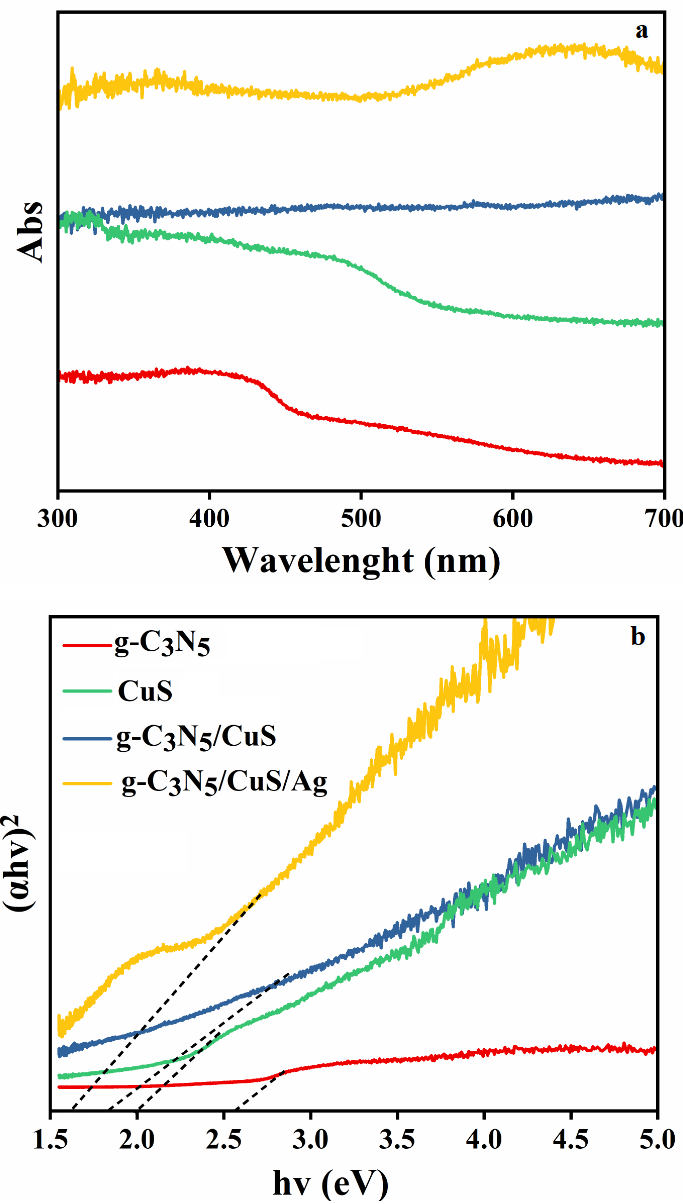


Figure S3 (a).UV-DRS and (b) Tauc plot


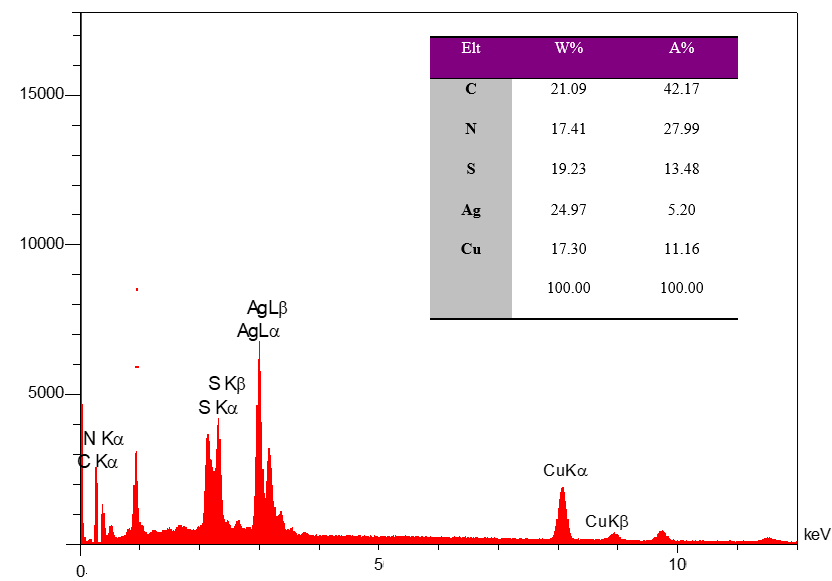


Figure S4.EDS analysis of g-C_3_N_5_/CuS/AgNPs composite


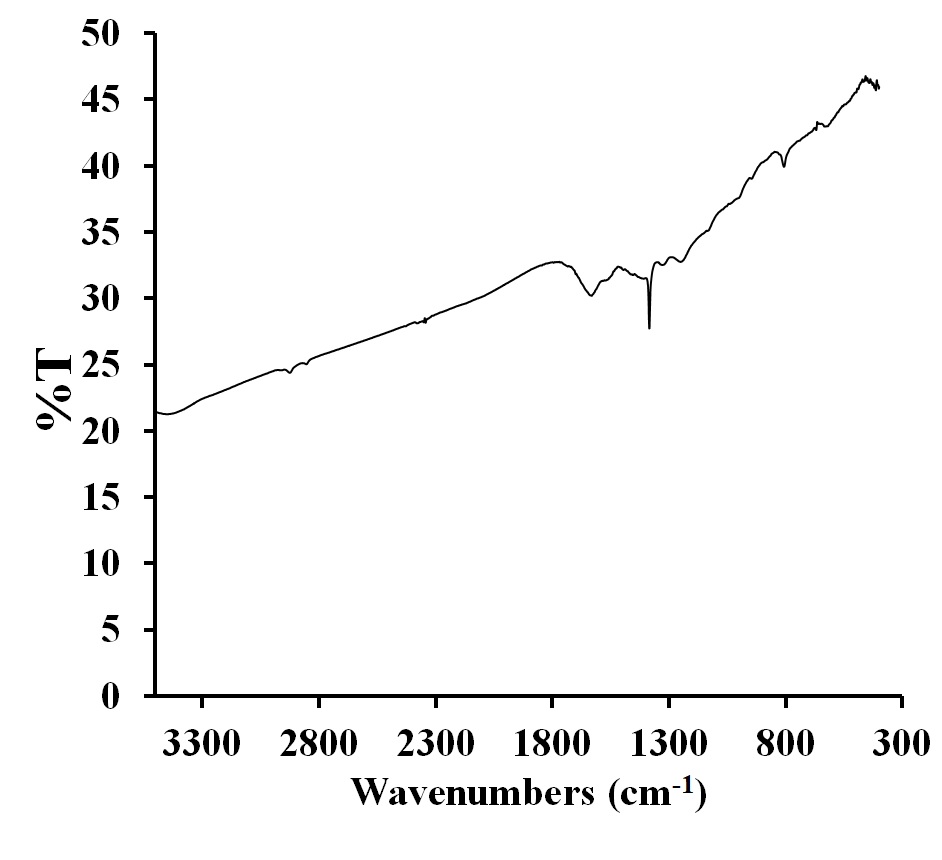


Figure S5.FT-IR of g-C_3_N_5_/CuS/AgNPs nanocomposite


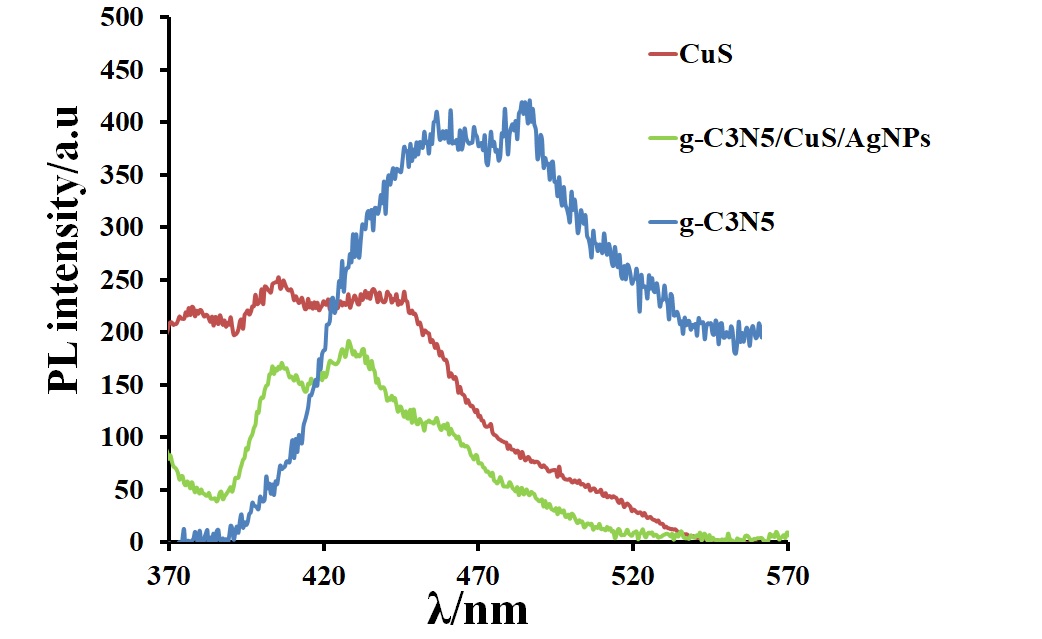


Figure S6. PL Spectra of synthesis of materials


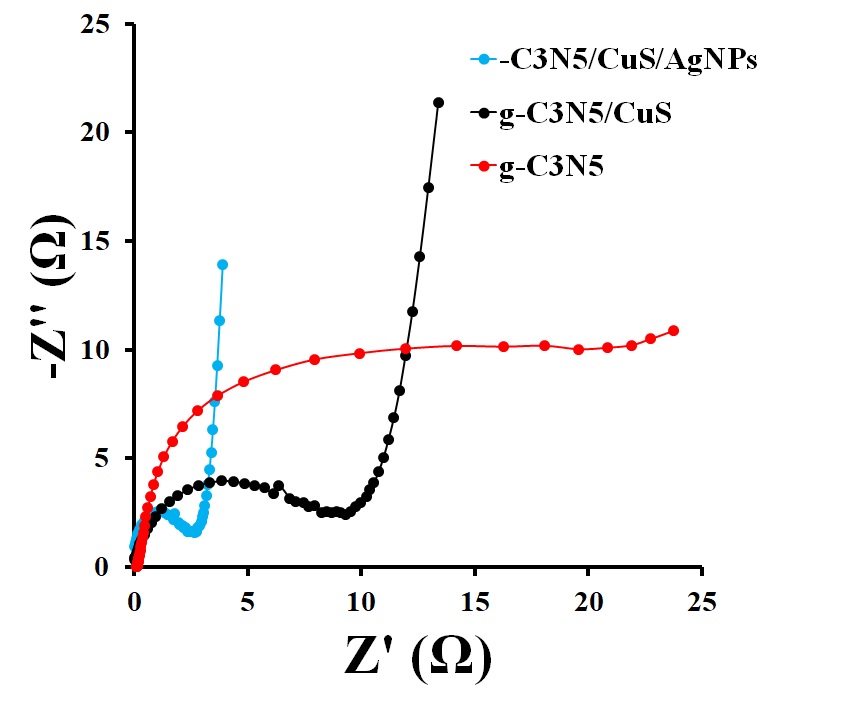


Figure S7. EIS Nyquist of synthesis of materials


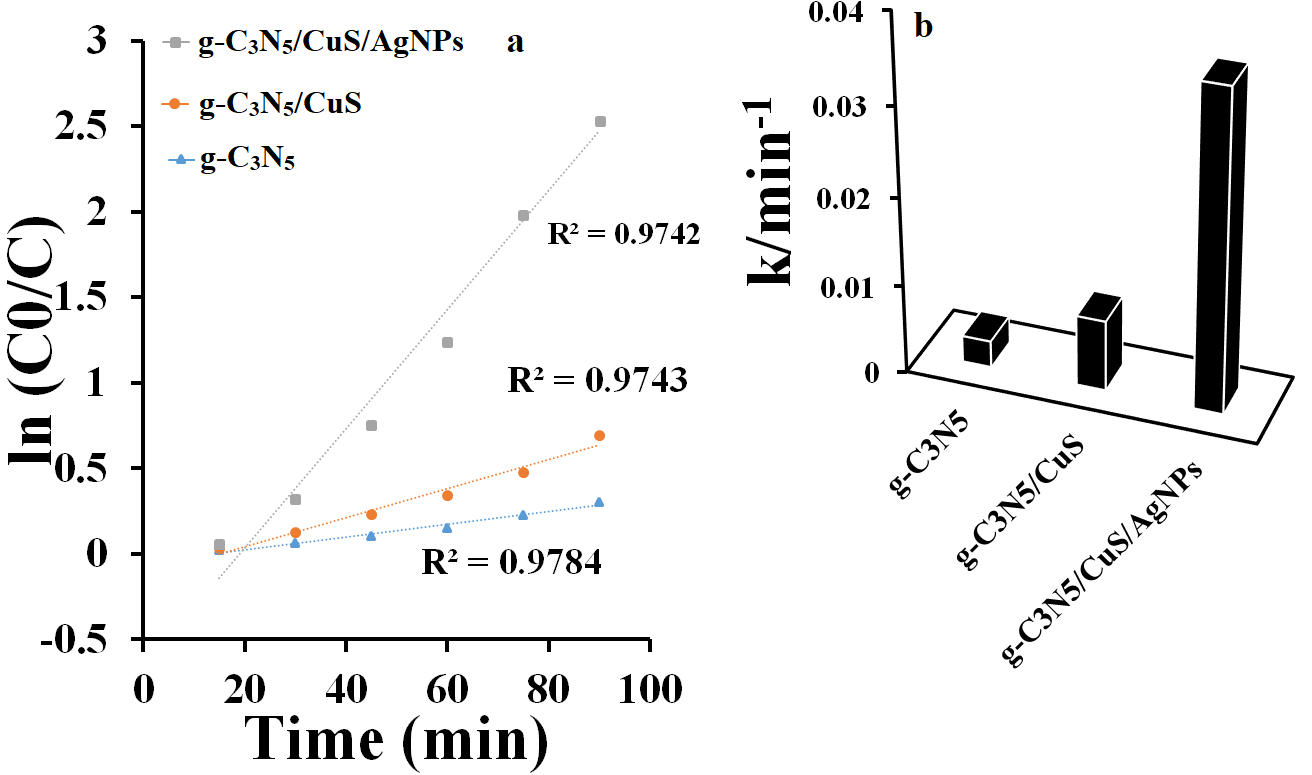


Figure S8. (a) The graph of the logarithmic changes in the initial concentration of parathion versus its concentration over different time (b) The kinetic degradation rate constant of parathion in the presence of synthesized materials

References

1. Nasiripur P, Zangiabadi M, Baghersad MH (2021) Visible light photocatalytic degradation of methyl parathion as chemical warfare agents simulant via GO-Fe3O4/Bi2MoO6 nanocomposite. Journal of Molecular Structure 1243:130875

2. Zangiabadi M, Mehrabi F, Nasiripur P, Baghersad MH (2022) Visible-light-driven photocatalytic degradation of methyl parathion as chemical warfare agent simulant by NiO/Bi2MoO6 heterojunction photocatalyst. Journal of Molecular Structure 1256:132472

3. Chen H, Shen M, Chen R, Dai K, Peng T (2011) Photocatalytic degradation of commercial methyl parathion in aqueous suspension containing La‐doped TiO2 nanoparticles. Environmental technology 32 (13):1515-1522

4. Zheng L, Pi F, Wang Y, Xu H, Zhang Y, Sun X (2016) Photocatalytic degradation of Acephate, Omethoate, and Methyl parathion by Fe3O4@ SiO2@ mTiO2 nanomicrospheres. Journal of hazardous materials 315:11-22

5. Aulakh MK, Kaur S, Pal B, Singh S (2020) Morphological influence of ZnO nanostructures and their Cu loaded composites for effective photodegradation of methyl parathion. Solid State Sciences 99:106045

6. Aghaei M, Sajjadi S, Keihan AH (2020) Sono-coprecipitation synthesis of ZnO/CuO nanophotocatalyst for removal of parathion from wastewater. Environmental Science and Pollution Research 27 (11):11541-11553

7. Rizo J, Díaz D, Reyes-Trejo B, Arellano-Jiménez MJ (2020) Cu2O nanoparticles for the degradation of methyl parathion. Beilstein Journal of Nanotechnology 11 (1):1546-1555

8. Singh AK, Ahlawat A, Dhiman TK, Lakshmi G, Solanki PR (2021) Degradation of methyl parathion using manganese oxide (MnO2) nanoparticles through photocatalysis. SGS-Engineering & Sciences 1 (01)

9. Rengaraj S, Li X, Tanner P, Pan Z, Pang G (2006) Photocatalytic degradation of methylparathion—an endocrine disruptor by Bi3+-doped TiO2. Journal of molecular catalysis A: chemical 247 (1-2):36-43
